# Investigation of the Trend in Adolescent Mental Health and its Related Social Factors: A Multi-Year Cross-Sectional Study For 13 Years

**DOI:** 10.3390/ijerph17155405

**Published:** 2020-07-27

**Authors:** Kyoung Min Kim, Dohyun Kim, Un Sun Chung

**Affiliations:** 1 Department of Psychiatry, College of Medicine, Dankook University, Cheonan 31116, Korea; profuture@naver.com; 2 Department of Psychiatry, Dankook University Hospital, Cheonan 31116, Korea; uptothebest@gmail.com; 3 Department of Psychiatry, Kyungpook National University Children’s Hospital, Daegu 41404, Korea

**Keywords:** adolescent, mental health, demographics, income inequality, academic performance

## Abstract

We aimed to investigate the mental health change and associated social correlates in adolescents in terms of depression, suicidal ideation, and suicidal attempt. In total, 978,079 students (12–18 years old, 7th–12th grade) participated in the survey for 13 years (2006–2018) by a multiple-year cross-sectional design (not a repeat measure for smaller group). Mental health outcome variables were assessed using self-report surveys with the independent variables of sex, age, economic status, school achievement, and parental educational level. Korean social indices of income inequality (Gini index, higher scores representing greater economic inequity with score range of 0–1), education (national proportion of tertiary education attainment), and actual suicides were investigated together as related social factors. The prevalence of depressive episodes, suicidal ideation, and suicidal attempt markedly dropped by 34.6%, 42.2%, and 48.3%, respectively. Moreover, the Gini index (from 0.314 in 2008 to 0.295 in 2015) and proportion of tertiary education (from 82.1% in 2006 to 69.7% in 2018) showed a decreasing tendency. These indices and adolescent mental health outcomes highly correlated with each other (Pearson’s r between Gini index and depressive episode = 0.789, suicidal ideation = 0.724, and suicidal attempt = 0.740; Pearson’s r between proportion of tertiary education and depressive episode = 0.930, suicidal ideation = 0.809, and suicidal attempt = 0.851). Adolescent mental health has improved in the last 13 years in Korea, and improvements in social inequality (decreased Gini index) and lessened burden of academic competition (decreased national proportion of tertiary education) were significantly associated with the improvement of adolescent’s mental health. However, the impact of parental educational level on children’s mental health was relatively minimal, compared with the impact of economic inequality and academic burden. Further studies are needed to reveal the underlying mechanism for the association between adolescent mental health and sociodemographic factors to save adolescents from psychological distress.

## 1. Introduction

Adolescence is a period wherein physical, cognitive, emotional, and social aspects drastically change in life [1,2]. While the transition from childhood to adolescence is defined by the biological changes during puberty, the transition from adolescence to adulthood is defined by numerous social tasks, such as family formation, education completion, and job employment [3]. Thus, adolescents are challenged by various developmental tasks, including school achievement, independency from parents, and peer relationship [4].

The rapid biological changes and developmental tasks during puberty make adolescents vulnerable to psychiatric disorders [5]. The prevalence of depression, which is low in children (under 1%) [6], rises substantially during adolescence [7]. A study reported that one-year point prevalence of overall depressive cases drastically increased from 1.1% at age 11 to 16.8% at age 18 [8]. Another school-based survey with 9863 U.S. youths aged 11–15 years reported that 18% of youths experienced the symptoms of depression (25% of female and 10% of male) [9]. A meta-analysis reported that the depression score assessed by Children’s Depression Inventory (CDI) increased by 12.8% from 8.8 at age 8 to 10.1 at age 16, especially in girls [10]. Mental disorders such as anxiety (31.9%) and mood disorder (14.3%) were reported to be highly prevalent among adolescents [11].

Accordingly, many Korean youths were also reported to experience depressive symptoms. Although there is a lack of longitudinal studies, previous studies have reported the differentially high prevalence of depressive symptoms in Korean adolescents, which suggests the change of the prevalence over the time. Suh and Cho (1997) [12] reported increased prevalence of adolescent depression in Korea, with 38% prevalence of depression in Korean adolescents. using the Center for Epidemiologic Studies Depression Scale (CES-D) [13], and they reported high prevalence rates of probable depression of 32.74%. A later study found 38% of probable depression (≥16 on CES-D score) and 21.2% of definite depression (≥25 on CES-D score) [14]. Another study conducted in 2008 reported the prevalence rates of probable depression (≥15 on CES-D score) of 27.2% and definite depression (≥21 on CES-D score) of 14.8% [15].

Increased concerns on the “epidemic” of child and adolescent depression have been published by general media on the basis of increases in antidepressant prescription and suicidal rates [16]. Alongside these concerns, a meta-analysis investigated the prevalence of child or adolescent depression over the past 30 years and reported no time effect for the prevalence between 1965 and 1996 [16]. However, repeated epidemiological cohorts reported the increasing rate of depression in adolescents [17]. These discrepant results of previous studies might be attributable to cultural differences or changes in social factors across the different time periods.

The etiology of mental disorders is complex and multifactorial; biologic vulnerability and individual stressful life events contribute to the occurrence of mental disorders [18,19]. However, mental disorders are also strongly influenced by social determinants, such as poor education, poverty, unemployment, national wealth, poor healthcare access, and social violence [20,21,22,23]. Notably, higher income inequality has been reported to affect negatively on the adolescents’ mental health [24,25]. In addition, academic stress is another potent environmental stressor affecting the adolescent’s mental health. Especially, adolescent in Asian countries have been reported to suffer more psychological distress from the academic stress than Western adolescents, due to the cultural tradition emphasizing the academic achievement of Asian countries [26]. Finally, low parental educational level is also a known risk factor for negative mental health of their offspring [27,28]. Thus, investigating the changing aspect of mental health among adolescents and these related social factors across a long-term period would contribute in revealing on how social factors affect the mental health of adolescents.

The Republic of Korea (South Korea) is one of the countries that experienced very rapid social changes over the past half-century. The gross domestic products (GDP) per capita soared by 25.6-fold from $1078 in 1960 to $27,578 in 2010, whereas the GDP per capita of the Unites States increased by 2.8-fold [29]. The average number of education years also increased from merely 4.1 years in 1960 to 12.0 years in 2010 [29]. In addition, the national proportion of tertiary education attainment reached 82.1% in 2005 [30], which implies very high academic pressure in adolescents. Meanwhile, the fertility rate sharply decreased from 4.5 children per woman in the 1970s to 1.19 in 2013, which is the lowest in the world [31]. While the rate of urbanization in Korea increased from 28.0% in 1960 to 81.5% in 2005 [32], the family size also decreased from 5.24 persons per family in 1970 to 3.12 persons in 2000 [33]. These marked and rapid changes in socioeconomic environment may affect the adolescents now growing up in Korea, including their mental health. Moreover, due to the worldwide financial crisis of 2007–2008, Korea also experienced the economic downturn and subsequent recovery [34]. A previous study reported that the suicidal rate in Korean adults increased during the recessionary period from 2009 to 2010 [35]. However, there is a lack of studies for the trends of mental health in adolescents, accompanied by these social changes over the time in Korea.

Thus, the present study aimed to investigate two research questions: (1) Was there any change in the prevalence of mental health problem in Korean adolescents between 2006 and 2018 in terms of depression, suicidal ideation, and suicidal attempt (2) Did the changes in the aforementioned social factors (income inequality, burden from academic achievement on adolescents, and parental educational level) have significant associations with change in mental health in adolescents? To answer these research questions, this study used the multi-year cross-sectional study design with a large sample of the Korean adolescent population between 2006 and 2018. This study might provide evidence on the changing aspect and related social factors of adolescent mental health.

## 2. Materials and Methods

### 2.1. Participants

This study analyzed data included in the Korean Youth Risk Behavior Web-based Survey (KYRBS) [36]. KYRBS is a national multi-year cross-sectional survey that has been conducted by the Korean Centers for Disease Control and Prevention (K-CDC) annually. KYRBS adopted the multi-stage cluster sampling design to obtain the representative sample of Korean adolescents. Among the target population of all public and private middle and high school students in 17 provinces in Korea, around 800 schools (400 middle and 400 high schools) were selected for the survey yearly, and all students in one sampled class per grade of every sampled school were invited to participate. The sampling plan has been designed every year since 2011 (every three years between 2005 and 2010). In total, 60,040–75,643 participants were invited annually in the survey. Among a target population of 960,100 adolescents, a total of 919,855 (95.8%) students aged 12–18 years belonging to 7th–12th grade agreed to participate from 2006 to 2018. Because the sampled classes or schools changed every year, the participating students were different individuals despite a rare possibility of duplication. The number of samples by academic year with the regional distribution is shown in Table S1. Among the total population, 51.6% of the participants were male with mean age of 15.46 (SD = 1.72) years. The study was performed using an anonymous questionnaire. KYRBS was aimed to assess the aspects of health risk behaviors in Korean youths, and it comprised 125 items including information on tobacco use, alcohol use, obesity, physical activity, sexual behavior, substance use, Internet use, and mental health. Some items included in the questionnaire changed slightly annually. However, the items included in the analysis in the present study remained the same from 2006 to 2018. Further information about the KYRBS survey design and methods is available from a published data resource profile [36]. Students who agreed to participate responded to an anonymous structured questionnaire presented on a computer screen.

### 2.2. Ethics Approval and Consent to Participate

Before participating in the survey, students were thoroughly informed about the purpose and process of the survey by a trained teacher and written informed consent was obtained from the students. The protocols of KYRBS were approved by the institutional review board of K-CDC. KYRBS has been performed by the government office as the national approved statistical data according to the national legislation (National Health Promotion Act). The need of ethics approval for the analysis protocol of the present study was waived by the institutional review board of Dankook University Hospital because the raw data are available without any identifying information.

### 2.3. Assessment

Participants were assessed by a self-report questionnaire provided on a computer screen. Dependent variables included the overall perceived happiness, depressive episode, suicidal ideation, and suicidal attempt in the last 12 months. The overall happiness was asked by one question, that is, “How happy do you usually feel?” to be answered using the five-point Likert scale (ranging from 1 to 5) with the responses of “very unhappy”, “unhappy”, “neutral”, “happy”, and “very happy”. A higher score represents a happier status. Meanwhile, the experiences of depressive episode, suicidal ideation, and suicidal attempt in the last 12 months were asked using one question with binary responses of yes or no as follows: “Did you experience sadness or desperation strong enough to stop your daily life for 2 weeks in the last 12 months?” for depression, “Did you think seriously about committing suicide in the last 12 months?” for suicidal ideation, and “Did you attempt suicide in the last 12 months?” for suicidal attempt.

The demographic factors such as sex, grade, perceived economic status, academic performance, and parental educational level constituted the independent variables predicting the mental health outcomes. The perceived economic status and school achievement were asked by one question to be answered using the five-point Likert scale.

### 2.4. Social Index

To examine the association of social index with the participants’ mental health outcome, we investigated two representative social indices: Gini index [37] and proportion of admission to tertiary education service [30] in South Korea during the study period. Developed by Corrado Gini in 1912, the Gini index is a widely used statistical measure of income inequality in a society [38]. The index ranges from 0 to 1, with higher scores representing greater inequality. In addition, the statistics of actual completed suicides in adolescents aged 15 to 19 were also examined to confirm the trend of the actual suicidality [39].

### 2.5. Statistical Analysis

The demographic variables and the change of prevalence in the adolescent mental health were analyzed by descriptive statistics. To examine the association between the subjective happiness and demographic variables, we employed the analysis of covariance (ANCOVA), with the subjective happiness regarded as a dependent variable of a continuous scale. The odds ratio (OR) for the depressive episode, suicidal ideation, and suicidal attempt according to the demographic variables were analyzed using the logistic regression model. The aforementioned ANCOVA and logistic regression were performed with two models separately. Model 1 used the independent variables such as sex, grade, economic status, and academic achievement as categorical scales. Model 2 included the paternal and maternal educational levels in addition to those in Model 1. To examine the association between the four social factors (i.e., Gini index and proportion of tertiary education) and the mental health outcomes from the survey, we calculated the Pearson correlation coefficients between the variables. Statistical analyses were conducted using the software package SPSS 25.0 for Windows (SPSS Inc., Chicago, IL, USA).

## 3. Results

### 3.1. Demographic Characteristics

Table 1 shows the demographic characteristics of participants. As depicted in the table, the total proportions of experiencing depressive episode, suicidal ideation, and suicidal attempt were 32.5%, 17.2%, and 4.0%, respectively, which is substantially high prevalence. In the demographic variables, the “middle” response in self-perception on household economic status and school achievement was the most prevalent, accounting for 47.2% and 27.5%, respectively. About 21% of participants regarded their household’s economic status as “low or low-middle”, while about 36% reported their school achievement as “low or low-middle”. This implies that the self-perception for their school achievement is lower than the household’s economic status. The “above 12 years” and “12 years” (graduated from high school) responses in the paternal educational level (42.9%) and the maternal educational level (42.2%) were the most prevalent, respectively, with a small proportion of “under 12 years” less than 5.0%.

### 3.2. Change of Prevalence for Mental Health Outcome Variables at Each Year

**Figure 1:**
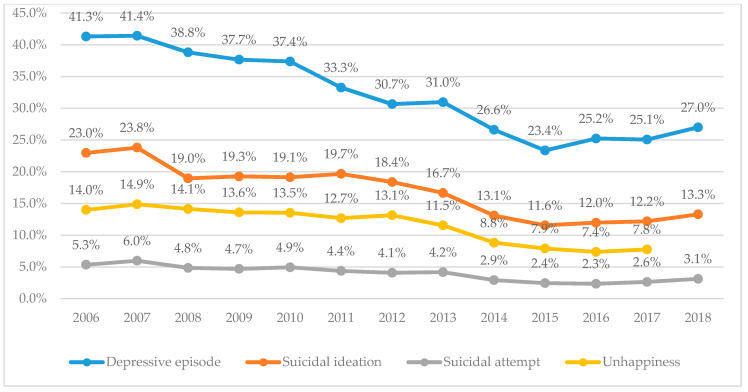
Changes of prevalence in adolescents’ mental health.

The yearly prevalence of mental health outcome variables such as the depressive episode, suicidal ideation, suicidal attempt, and subjective unhappiness proportion tended to considerably drop during the study period despite a slightly increasing trend since 2015 (Figure 1 and Tables S2 and S3). These findings indicate the overall improvement of adolescent mental health during the study years.

### 3.3. Association Between the Mental Health Outcomes and the Demographic Variables

Table 2 and Table S4 present the association between subjective happiness and each of the demographic variables. Among all variables, economic status had the largest effect size for subjective happiness (F = 7936, *p* < 0.001, partial η^2^ = 0.0356), followed by academic achievement (F = 2748, *p* < 0.001, partial η^2^ = 0.0122). Although the associations between parental educational level and subjective happiness were significant, the effect sizes were minimal (F = 49, *p* < 0.001, partial η^2^ = 0.0002 for paternal education; F = 116, *p* < 0.001, partial η^2^ = 0.0004 for maternal education). The participants with higher economic status, academic achievement, and parental educational level showed the higher scores on the subjective happiness (Table S4). Male and younger participants were happier than female and older ones.

Table 3 and Table S5 show the OR for the depressive episode, suicidal ideation, and suicidal attempt based on the demographic variables. Females had higher ORs of all these outcomes than males. In older age, the OR significantly increased for depressive episode but significantly decreased for suicidal attempt.

Adolescents with “low-middle” and “low” economic statuses had significantly increased OR for depressive episode (OR = 1.26 and 1.80, respectively) and suicidal ideation (OR = 1.36 and 2.13, respectively), which implies the negative association between economic status and the mental health in participants. Likewise, adolescents with “low-middle” and “low” academic achievements showed increased OR for depressive episode (OR = 1.39 and 1.71, respectively), suicidal ideation (OR = 1.24 and 1.55, respectively), and suicidal attempt (OR = 1.31 and 1.97, respectively), representing the association between low academic achievement and negative mental health. The paternal educational level under 12 years had a significantly increased OR of 1.15 for suicidal attempt only, whereas the maternal educational level under 12 years had significantly increased ORs for depressive episode (OR = 1.13), suicidal ideation (OR = 1.16), and suicidal attempt (OR = 1.15).

### 3.4. Association between the Change in Mental Health Outcome and Korean Social Index

**Figure 2:**
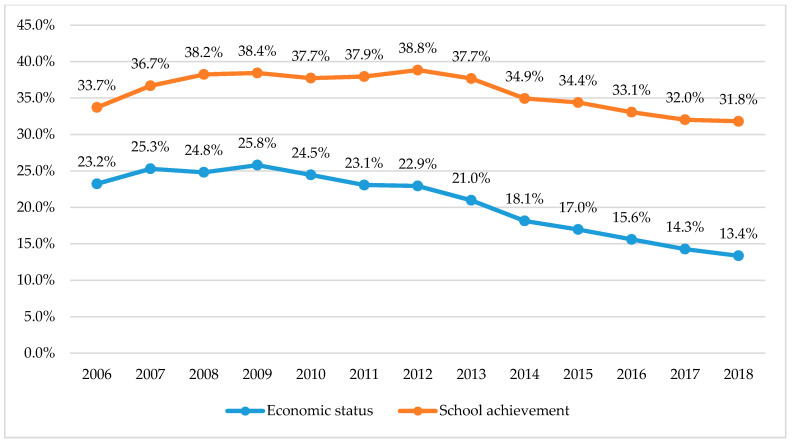
Changes in adolescents’ perceived economic status and school achievement. Changes in the proportion of “middle-low and low” responses to economic status and school achievement. Economic status: Annual proportion of participants who responded “low middle” plus “low” among all participants. School achievement: Annual proportion of participants who responded “low middle” plus “low” among all participants.

**Figure 3:**
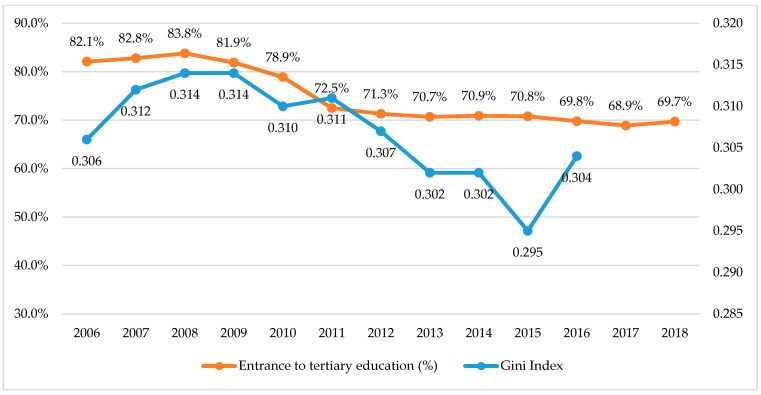
Changes in social index related to economic status and school achievement.

Table S6 and Figure 2 show the decreasing trend of proportional changes in “low-middle and low” responses to economic status and school achievement, representing the overall improvement on the self-perception of the adolescents for the economic status and school achievement. Meanwhile, Table S7 and Figure 3 present the change of Korean social index, which comprised the Gini index and proportion of admission to tertiary education service. The results of the Gini index and proportion of admission to tertiary education service also showed the decreasing tendency.

**Figure 4:**
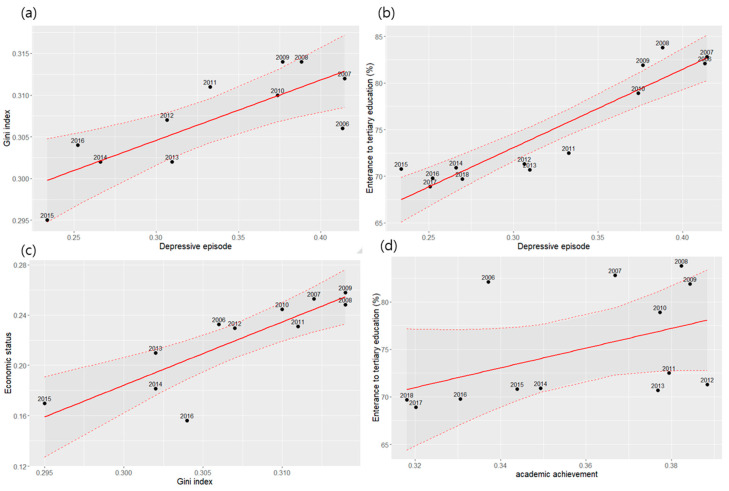
Correlation of the changes between social index and survey responses between 2005 and 2018. Scatter plots showing the correlation between: the Gini index and depressive episode (**a**); proportion of admission to tertiary education service and depressive episode (**b**); Gini index and economic status (**c**); and proportion of admission to tertiary education service and academic achievement (**d**). The dashed line represents 95% confidence interval of the regression line.

Table 4 and Figure 4 depict the correlation among the yearly assessments of Korean social index and demographic and outcome variables of our data. The four mental health outcome variables and economic status and school achievement had a significantly high correlation coefficient with the Gini index and proportion of tertiary education, which suggest the substantially high association between these social factors and the mental health outcomes in adolescents. The trend of actual completed suicidality in Korean adolescents presented substantially decreasing trend with the peak in 2009, in accordance with our findings for the suicidal ideation and attempt.

## 4. Discussion

Korea has the highest suicidal rate of the overall population among the Organization for Economic Cooperation and Development (OECD) countries from 2003 to 2016 [40]. Suicide is also the primary cause of death among Korean adolescents [41]. Fortunately, the overall suicidal rate has been decreasing, with the peak in 2009 [42]. The first finding of our study is the dropping rate of negative mental health outcomes such as unhappiness, depressive episode, suicidal ideation, and suicidal attempt. In a previous meta-analysis, the prevalence of child or adolescent depression had not significantly changed for 30 years [16]. In addition, a study with the US population reported increasing prevalence of adolescent depression from 2005 to 2014 [43]. The present findings for the decreasing trend of adolescent depression are encouraging, warranting further exploration of the associated factors that might contribute to adolescent mental health.

### 4.1. Economic Inequality

The effect size (partial eta square) of economic status as a predictor for subjective happiness was the largest among all the independent variables. Furthermore, the OR of economic status for depressive episode and suicidal ideation was the highest among all the factors, especially in the “low” group. Poverty and income inequality are widely accepted to have a negative effect on the mental health of adolescents as well as adults [21,44,45]. The present findings implicate an impact of economic status on the mental health of youth, which are consistent with previous studies.

In addition, the prevalence of depressive episode and suicidal ideation decreased accordingly, with a markedly decreasing proportion of participants who reported the “low and middle-low” in economic status from 2006 to 2018. Thus, the correlation coefficient between the yearly proportion of “low and middle-low” group in economic status and the prevalence of depressive episode and suicidal ideation was substantially high. Although the macroeconomic decline at the national level was reportedly associated with an increased risk of depression and anxiety [46], the impact of rapid economic change on the burden caused by mental disorders remains uncertain [45]. Our findings imply that adolescent mental health could be improved with the decrease of economic inequality.

However, these findings could be biased because economic status was only assessed by participants’ self-report. Although adolescent mental disorders are related to the perceived social status rather than the objective socioeconomic status [47], we investigated the Gini index of Korea to confirm the association between real economic inequality and participants’ mental health. In our study, the Gini index also tended to drop, and the correlation between the Gini index and prevalence of depressive episode was as high as 0.789. These findings confirm the association between the economic inequality and mental health of the adolescents.

However, we should be cautious to interpret the association between the Gini index and adolescent mental health as a linear one. The association between the Gini index and social happiness was previously presented as an inverted U-shaped curve, with the inflection points of 0.301 and 0.306 [48]. In other words, Gini index (income inequality) is negatively associated with social happiness in the area over the inflection points (about 0.30). However, in the area under the inflection points, Gini index is positively associated with social happiness. These inflection points in Gini index from the previous study are similar to the Gini index from our study (0.295–0.314). In addition, income inequality has the differential associations with social happiness in low- and high-income countries [49]. Income inequality has a negative impact on happiness in high-income countries. Conversely, in poor countries, with below $20,000–30,000 per capita income, income inequality was reported to raise happiness rather than lower it. Korean GDP per capita increased from $18,291 in 2009 to $31,362 in 2018 [50]. Korea seems to be near the inflection point for the association among social wealth, inequality, and adolescent mental health in terms of economic distribution and gross national wealth.

Notably, the ORs for depressive episode, suicidal ideation, and suicidal attempt were only higher in “low” and “low-middle” groups. A previous study reported that the risk of mental health problems was 2–3 times higher in socioeconomically disadvantaged adolescents [51]. Our findings suggest economic deprivation as a risk factor of mental health problem. Hence, additional social attention and support are needed for highly deprived adolescents in terms of economic status.

### 4.2. Academic Achievement

In our study, the effect size of school performance for the adolescents’ subjective happiness was the second highest following economic status. Adolescents in Asian countries such as China, Taiwan, Singapore, Japan, and Korea, which possess Confucian Heritage Culture, have high academic expectations from parents and feel that high academic achievement is their obligation to live up to the expectation of parents [26]. Thus, low academic performance often leads to negative mental health. A cross-cultural study reported that Singaporean adolescents suffered from a significantly higher level of academic stress arising from the expectations of self and other people compared to their Canadian counterparts [52].

The national proportion of tertiary education, which refers to the proportion of students who entered into any type of education beyond the high school level, in Korea ranks the highest among OECD countries, at 70% in 2018 [53]. Actually, Korean adolescents were reported to experience severe distress from long hours of study, which is called “examination hell” [54]. Another study also reported that more than half of Korean adolescents (54.7%) chose the “worry for the schoolwork” as their biggest stressor among the various stressful life events [55], These previous findings suggest immensely high social emphasis on education, a strong competition level in the academic performance, and a big burden that Korean adolescents feel from the academic performance to enter into the better tertiary school.

However, in our study, the proportion of tertiary education tended to drop substantially. Furthermore, the decreasing tendency in proportion of tertiary education presented highly positive correlation with the decreased depressive episode and suicidal ideation. The admission capacity of tertiary education in Korea is already over the number of high school graduates. Thus, a decreased proportion of tertiary education might indicate that the voluntary non-entrance to tertiary education increased and the degree of competition in academic achievement decreased, although still high. Moreover, in our study, the proportion of participants who reported their academic achievement as “middle-low and low” also decreased from 38.2% in 2008 to 31.8% in 2018. Although the findings are hypothetical, they suggest that the overall level of academic competition was alleviated, which contributed to the improvement of the relative self-perception on academic achievement and overall mental health in adolescents. Further studies are warranted to explore the underlying mechanism for the association among the proportion of tertiary education, self-perception of school achievement, and mental health in adolescents observed in our study.

### 4.3. Parental Education

Lower educational levels of parents are well-known risk factors for children’s negative mental health [27,51,56]. However, the impact of parental educational level on children’s mental health (assessed by the effect size and OR) was relatively minimal in our study, compared with the impact of economic status or school achievement. The results from previous studies regarding the impact of parental educational level on children’s mental health are inconsistent with our study. Studies conducted in German [57], Spanish [27], and US [47] populations reported that parental education, rather than the household income, is the strongest risk factor for children’s mental health problem. A US study reported that a low parental educational level significantly predicted the persistence and severity of children’s mental disorder, not the onset, whereas financial problem was related to the onset of children’s mental disorder [56]. These discrepancies might arise by sociocultural differences or may reflect disparate findings from a paucity of studies. Hence, further investigation, such as cross-cultural comparison, is encouraged. Furthermore, our study presented that the maternal educational level had a relatively larger effect on adolescent mental health than the paternal educational level, consistent with previous studies [27,58], suggesting the importance of improving maternal education.

### 4.4. Limitations

This study has some noteworthy limitations. Although perceived social status is more related to the adolescent’s mental disorder [47], the objective aspect of socioeconomic status could affect the mental health of adolescents. However, our study was only based on the self-report of adolescents and did not include reports from parents. In addition, missing data for the parental educational level were considerably numerous. The four outcome variables, namely, subjective happiness, depressive episode, suicidal ideation, and suicidal attempt, were also assessed simply, with only one question each. These simplified assessments might possibly cause the validity problems of assessing those psychological constructs, including unfaithful responses from the adolescents due to anonymous questionnaire and recall biases for the responses to experience in the last one year. Although the previously reported high prevalence of depression among Korean youths [14,15], corresponding to our study finding, mitigates the concerns related to the validity of assessment, future studies with validated assessment tools and detailed information from multiple informants are needed to confirm the present findings. Second, educational policy implemented by the government during the study period, as well as the social factors, could affect the improvement of adolescent mental health. Unfortunately, we did not consider these policy factors. Third, although we tried to include various independent variables, including sex, age, income inequality, school achievement, and parental educational level as well as social indices, we did not consider all the variables included in the KYRBS [36] for the analysis. Moreover, we failed to include individual variables, such as stressful life events and parental psychopathology, which are importantly related to mental health. These realizations limit the generalizability of our findings. Hence, future studies should consider these factors altogether to help improve adolescent mental health.

## 5. Conclusions

The present findings revealed the changing trend of adolescent mental health in Korea for the last 13 years and suggested that income inequality and school achievement are the related social factors. Although our study provides important clues to improve adolescent mental health, more elaborate studies are necessary to confirm the present findings.

## Figures and Tables

**Table 1:** Demographic characteristics.

	Unweighted *n* (%)
Total	919,855 (100.0)
Male	474,264 (51.6)
Female	445,591 (48.4)
Age; mean (SD)	15.46 (1.72)
Grade	
7th (Middle school 1st)	154,385 (16.8)
8th (Middle school 2nd)	155,759 (16.9)
9th (Middle school 3rd)	157,107 (17.1)
10th (High school 1st)	152,425 (16.6)
11th (High school 2nd)	151,834 (16.5)
12th (High school 3rd)	148,345 (16.1)
Economic status	
High	69,370 (7.5)
High middle	223,162 (24.3)
Middle	434,453 (47.2)
Low middle	148,476 (16.1)
Low	44,394 (4.8)
Academic achievement	
High	111,030 (12.1)
High middle	224,742 (24.4)
Middle	253,275 (27.5)
Low middle	224,026 (24.4)
Low	106,782 (11.6)
Paternal educational level	
<12 years	44,838 (4.9)
12 years	306,340 ((33.3)
>12 years	394,845 (42.9)
Unknown	173,882 (18.9)
Maternal educational level	
<12 years	45,177 (4.9)
12 years	387,817 (42.2)
>12 years	316,408 (34.4)
Unknown	170,453 (18.5)
Depressive episode	
Yes	299,056 (32.5)
No	620,796 (67.5)
Suicidal ideation	
Yes	158,193 (17.2)
No	761,659 (82.8)
Suicidal attempt	
Yes	37,201 (4.0)
No	882,654 (96.0)

**Table 2:** Association between subjective happiness and the demographic variables.

Variables	Model 1 (*n* = 859.815)			Model 2 (*n* = 651.533)		
	F	*p*	Partial η^2^	F	*p*	Partial η^2^
Economic status	7936	<0.001	0.0356	5180	<0.001	0.0308
Academic achievement	2662	<0.001	0.0122	2110	<0.001	0.0128
Sex	2748	<0.001	0.0032	1565	<0.001	0.0024
Grade	553	<0.001	0.0032	332	<0.001	0.0025
Paternal educational level				49	<0.001	0.0002
Maternal educational level				116	<0.001	0.0004

Model 1: Sex, grade, economic status, and academic achievement were included as covariates. Model 2: Paternal and maternal educational levels were included as covariates in addition to those in Model 1.

**Table 3:** Odds ratio for the depressive episode, suicidal ideation, and suicidal attempt.

	Depressive Episode	Suicidal Ideation	Suicidal Attempt
Sex ^a^			
Male	referent	referent	referent
Female	1.63 *	1.71 *	1.77 *
Grade ^a^			
7th	referent	referent	referent
8th	1.10 *	1.05 *	1.00
9th	1.20 *	1.04 *	0.92 *
10th	1.25 *	0.95 *	0.77 *
11th	1.32 *	0.95 *	0.70 *
12th	1.45 *	0.91*	0.63 *
Economic status ^a^			
High	referent	referent	referent
High middle	0.92 *	0.88 *	0.63 *
Middle	0.89 *	0.87 *	0.58 *
Low middle	1.26*	1.36 *	0.89 *
Low	1.80*	2.13 *	1.77 *
Academic achievements ^a^			
High	referent	referent	referent
High middle	1.08 *	0.98	0.86 *
Middle	1.16 *	1.01	0.99
Low middle	1.39 *	1.24 *	1.31 *
Low	1.71*	1.55*	1.97 *
Paternal educational level ^b^			
>12 years	referent	referent	referent
12 years	0.93 *	0.93 *	0.98
<12 years	1.00	1.03	1.15 *
Maternal educational level ^b^			
>12 years	referent	referent	referent
12 years	1.02	1.00	0.99
<12 years	1.13 *	1.16*	1.15*

* *p* < 0.001; ^a^ Model 1 with sex, grade, economic status, and academic achievement as covariates; ^b^ Model 2 with paternal and maternal educational levels as covariates in addition to those in Model 1.

**Table 4:** Correlation of the changes between social index and survey responses between 2006 and 2018.

	*n* = 13 (between 2006 and 2018)	Depressive Episode	Suicidal Ideation	Suicidal Attempt	Subjective Unhappiness	Economic Status	School Achievement
Social index	Gini index	0.789 **	0.724 *	0.740 **	0.798 **	0.845 **	0.616 *
	Tertiary education (%)	0.930 **	0.809 **	0.851 **	0.813 **	0.812 **	0.453
Subjective response	Economic status	0.880 **	0.882 **	0.890 **	0.972 **	1	0.852 **
	School achievement	0.542	0.582 *	0.608 *	0.724 **	0.852 **	1

Correlation coefficients were calculated with the annual data for each variable. Depressive episode: Annual proportion of “yes” response to the item of depressed mood. Suicidal ideation: Annual proportion of “yes” response to the item of suicidal ideation. Suicidal attempt: Annual proportion of “yes” response to the item of suicidal attempt. Unhappiness: Annual proportion of participants who responded “slightly unhappy” plus “very unhappy” among all participants. Economic status: Annual proportion of participants who responded “low middle” plus “low” among all participants. School achievement: Annual proportion of participants who responded “low middle” plus “low” among all participants. * *p* < 0.05, ** *p* < 0.01.

## Supplementary Materials

The following are available online at https://www.mdpi.com/1660-4601/17/15/5405/s1. Table S1: The number of participants by academic years with the regional distribution; Table S2: General perceived subjective happiness of participants in each year; Table S3: Distribution of depressive episode, suicidal ideation, and suicidal attempt; Table S4: Mean scores of subjective happiness according to the demographic factors (mean; SD); Table S5: Logistic regression analysis for depressive episode, suicidal ideation, and suicidal attempt with the demographic variables; Table S6: Distribution of perceived economic status and school achievement in each year; Table S7: Social index of South Korea during the study year.
