# Supplementary material for: Investigation of the Trend in Adolescent Mental Health and its Related Social Factors: A Multi-Year Cross-Sectional Study For 13 Years
Source: Int J Environ Res Public Health. 2020 Jul 27;17(15):5405. doi: 10.3390/ijerph17155405 (PMC7432642; doi:10.3390/ijerph17155405)
Supplement: Supplementary file 1 [file ijerph-17-05405-s001.zip › ijerph-850326-supplementary.docx]

Supplementary Materials: Investigation of the Trend in Adolescent Mental Health and its Related Social Factors: A Multi-Year Cross-Sectional Study For 13 Years

**Table S1.** The number of participants by academic years with the regional distribution.

|  | Total (N) | Total (%) | 2006 | 2007 | 2008 | 2009 | 2010 | 2011 | 2012 | 2013 | 2014 | 2015 | 2016 | 2017 | 2018 |
| --- | --- | --- | --- | --- | --- | --- | --- | --- | --- | --- | --- | --- | --- | --- | --- |
| Gangwon-do | 36762 | 4.0% | 3402 | 3352 | 3520 | 3553 | 3447 | 2722 | 2486 | 2457 | 2669 | 2449 | 2224 | 2219 | 2262 |
| Gyeonggi-do | 160025 | 17.4% | 8291 | 8761 | 9332 | 9430 | 9044 | 14427 | 15658 | 15319 | 15158 | 14352 | 13990 | 13465 | 12798 |
| Gyeongsangnam-do | 60252 | 6.6% | 4848 | 5014 | 4849 | 4933 | 4810 | 4733 | 4920 | 4744 | 4737 | 4581 | 4132 | 4067 | 3884 |
| Gyeongsangbuk-do | 50996 | 5.5% | 4266 | 4497 | 4589 | 4589 | 4515 | 4028 | 3755 | 3924 | 3765 | 3557 | 3335 | 3112 | 3064 |
| Gwangju | 43007 | 4.7% | 3846 | 4076 | 4011 | 4110 | 4024 | 3309 | 3149 | 3002 | 3072 | 2850 | 2676 | 2441 | 2441 |
| Daegu | 55376 | 6.0% | 4953 | 5163 | 5053 | 5063 | 5030 | 4439 | 4312 | 4151 | 3952 | 3614 | 3292 | 3288 | 3066 |
| Daejeon | 41380 | 4.5% | 3798 | 3870 | 4011 | 3948 | 3874 | 3003 | 2975 | 2947 | 2966 | 2656 | 2682 | 2377 | 2273 |
| Busan | 61936 | 6.7% | 5195 | 5530 | 5501 | 5468 | 5303 | 5157 | 4939 | 4649 | 4436 | 4209 | 3979 | 3752 | 3818 |
| Seoul | 122614 | 13.3% | 7855 | 8402 | 8077 | 8207 | 7848 | 11355 | 11373 | 11165 | 10997 | 9710 | 9567 | 9287 | 8771 |
| Sejong | 3823 | 0.4% | 0 | 0 | 0 | 0 | 0 | 0 | 0 | 0 | 0 | 935 | 1012 | 932 | 944 |
| Ulsan | 36824 | 4.0% | 3561 | 3723 | 3665 | 3635 | 3478 | 2913 | 2518 | 2486 | 2449 | 2218 | 2114 | 2121 | 1943 |
| Incheon | 56853 | 6.2% | 4879 | 5122 | 5240 | 5126 | 5008 | 4516 | 4302 | 4140 | 4120 | 3847 | 3724 | 3431 | 3398 |
| Jeollanam-do | 40673 | 4.4% | 3564 | 3661 | 3581 | 3381 | 3381 | 3434 | 2906 | 2871 | 3111 | 2925 | 2820 | 2583 | 2455 |
| Jeollabuk-do | 42015 | 4.6% | 3336 | 3548 | 3757 | 3799 | 3778 | 3382 | 3133 | 3183 | 3012 | 2931 | 3060 | 2611 | 2485 |
| Jeju-do | 25115 | 2.7% | 2562 | 2638 | 2485 | 2336 | 2329 | 1795 | 1743 | 1560 | 1664 | 1597 | 1478 | 1476 | 1452 |
| Chungcheongnam-do | 43493 | 4.7% | 3624 | 3740 | 3910 | 3852 | 3869 | 3457 | 3264 | 3112 | 3216 | 3095 | 2990 | 2745 | 2619 |
| Chungcheongbuk-do | 38711 | 4.2% | 3424 | 3601 | 3657 | 3636 | 3500 | 2973 | 2753 | 2725 | 2736 | 2517 | 2453 | 2369 | 2367 |
| Total | 919855 | 100.0% | 71404 | 74698 | 75238 | 75066 | 73238 | 75643 | 74186 | 72435 | 72060 | 68043 | 65528 | 62276 | 60040 |

**Table S2.** General perceived subjective happiness of participants at each year.

| **Unweighted** n (%) | 2006 | 2007 | 2008 | 2009 | 2010 | 2011 | 2012 | 2013 | 2014 | 2015 | 2016 | 2017 |
| --- | --- | --- | --- | --- | --- | --- | --- | --- | --- | --- | --- | --- |
| Very happy | 12064 | 11519 | 12451 | 12590 | 12993 | 13629 | 13771 | 15410 | 17943 | 18276 | 18992 | 18573 |
|  | 16.9% | 15.4% | 16.5% | 16.8% | 17.7% | 18.0% | 18.6% | 21.3% | 24.9% | 26.9% | 29.0% | 29.8% |
| Slightly happy | 25705 | 26397 | 27039 | 26882 | 26732 | 29037 | 27941 | 26717 | 27853 | 26473 | 24964 | 23564 |
|  | 36.0% | 35.3% | 35.9% | 35.8% | 36.5% | 38.4% | 37.7% | 36.9% | 38.7% | 38.9% | 38.1% | 37.8% |
| Neutral | 23646 | 25669 | 25109 | 25387 | 23601 | 23387 | 22725 | 21943 | 19908 | 17922 | 16743 | 15309 |
|  | 33.1% | 34.4% | 33.4% | 33.8% | 32.2% | 30.9% | 30.6% | 30.3% | 27.6% | 26.3% | 25.6% | 24.6% |
| Slightly unhappy | 8214 | 9081 | 8736 | 8389 | 8221 | 8112 | 8310 | 7095 | 5403 | 4532 | 4102 | 4041 |
|  | 11.5% | 12.2% | 11.6% | 11.2% | 11.2% | 10.7% | 11.2% | 9.8% | 7.5% | 6.7% | 6.3% | 6.5% |
| Very unhappy | 1775 | 2032 | 1903 | 1818 | 1691 | 1478 | 1439 | 1270 | 953 | 840 | 727 | 789 |
|  | 2.5% | 2.7% | 2.5% | 2.4% | 2.3% | 2.0% | 1.9% | 1.8% | 1.3% | 1.2% | 1.1% | 1.3% |

**Table S3.** Distribution of depressive episode, suicidal ideation, and suicidal attempt.

| Unweighted n (%) | 2006 | 2007 | 2008 | 2009 | 2010 | 2011 | 2012 | 2013 | 2014 | 2015 | 2016 | 2017 | 2018 |
| --- | --- | --- | --- | --- | --- | --- | --- | --- | --- | --- | --- | --- | --- |
| Depressive episode | |  |  |  |  |  |  |  |  |  |  |  |  |
| Yes | 29498 | 30951 | 29202 | 28273 | 27373 | 25161 | 22745 | 22430 | 19174 | 15894 | 16535 | 15612 | 16208 |
|  | 41.3% | 41.4% | 38.8% | 37.7% | 37.4% | 33.3% | 30.7% | 31.0% | 26.6% | 23.4% | 25.2% | 25.1% | 27.0% |
| No | 41903 | 43747 | 46036 | 46793 | 45865 | 50482 | 51441 | 50005 | 52886 | 52149 | 48993 | 46664 | 43832 |
|  | 58.7% | 58.6% | 61.2% | 62.3% | 62.6% | 66.7% | 69.3% | 69.0% | 73.4% | 76.6% | 74.8% | 74.9% | 73.0% |
| Suicidal ideation | |  |  |  |  |  |  |  |  |  |  |  |  |
| Yes | 16397 | 17783 | 14259 | 14458 | 14011 | 14875 | 13635 | 12070 | 9438 | 7862 | 7845 | 7584 | 7976 |
|  | 23.0% | 23.8% | 19.0% | 19.3% | 19.1% | 19.7% | 18.4% | 16.7% | 13.1% | 11.6% | 12.0% | 12.2% | 13.3% |
| No | 55004 | 56915 | 60979 | 60608 | 59227 | 60768 | 60551 | 60365 | 62622 | 60181 | 57683 | 54692 | 52064 |
|  | 77.0% | 76.2% | 81.0% | 80.7% | 80.9% | 80.3% | 81.6% | 83.3% | 86.9% | 88.4% | 88.0% | 87.8% | 86.7% |
| Suicidal attempt | |  |  |  |  |  |  |  |  |  |  |  |  |
| Yes | 3817 | 4460 | 3649 | 3513 | 3616 | 3304 | 3018 | 3021 | 2104 | 1662 | 1530 | 1634 | 1873 |
|  | 5.3% | 6.0% | 4.8% | 4.7% | 4.9% | 4.4% | 4.1% | 4.2% | 2.9% | 2.4% | 2.3% | 2.6% | 3.1% |
| No | 67587 | 70238 | 71589 | 71553 | 69622 | 72339 | 71168 | 69414 | 69956 | 66381 | 63998 | 60642 | 58167 |
|  | 94.7% | 94.0% | 95.2% | 95.3% | 95.1% | 95.6% | 95.9% | 95.8% | 97.1% | 97.6% | 97.7% | 97.4% | 96.9% |

**Table S4.** Mean scores of subjective happiness according to the demographic factors (mean; SD).

| Demographic variables |  |  |  |  |  |  |
| --- | --- | --- | --- | --- | --- | --- |
|  | High | High-middle | Middle | Low-middle | Low |  |
| Economic status | 4.07 (1.00) | 3.87 (0.92) | 3.63 (0.92) | 3.36 (0.98) | 3.09 (1.14) |  |
| Academic achievement | 3.91 (0.97) | 3.77 (0.93) | 3.68 (0.93) | 3.53 (0.97) | 3.33 (1.07) |  |
| Sex | Male | Female |  |  |  |  |
|  | 3.71 (0.98) | 3.58 (0.97) |  |  |  |  |
| Grade | 7th | 8th | 9th | 10th | 11th | 12th |
|  | 3.81 (0.99) | 3.70 (0.99) | 3.67 (0.97) | 3.60 (0.97) | 3.55 (0.95) | 3.56 (0.96) |
| Parental educational level | <12 years | 12 years | >12 years |  |  |  |
| Paternal educational level | 3.46 (1.00) | 3.61 (0.96) | 3.73 (0.97) |  |  |  |
| Maternal educational level | 3.46 (0.98) | 3.61 (0.96) | 3.76 (0.97) |  |  |  |

**Table S5.** Logistic regression analysis for depressive episode, suicidal ideation, and suicidal attempt with the demographic variables.

|  | Model 1 (n = 919855) | | | Model 2 (n = 694753) | | |
| --- | --- | --- | --- | --- | --- | --- |
|  | Depressive episode | Suicidal ideation | Suicidal attempt | Depressive episode | Suicidal ideation | Suicidal attempt |
| Sex |  |  |  |  |  |  |
| Male | referent | referent | referent | referent | referent | referent |
| Female | 1.63 (1.61–1.64) | 1.71 (1.69–1.73) | 1.77 (1.74–1.81) | 1.58 (1.57–1.6) | 1.67 (1.65–1.70) | 1.75 (1.70–1.79) |
| Grade |  |  |  |  |  |  |
| 7^th^ | referent | referent | referent | referent | referent | referent |
| 8^th^ | 1.10 (1.09–1.12) | 1.05 (1.03–1.07) | 1.00 (0.96–1.03) | 1.08 (1.06–1.11) | 1.01 (0.99–1.03) | 0.92 (0.89–0.96) |
| 9^th^ | 1.20 (1.18–1.22) | 1.04 (1.02–1.06) | 0.92 (0.89–0.95) | 1.18 (1.16–1.20) | 1.00 (0.98–1.02) | 0.86 (0.82–0.89) |
| 10^th^ | 1.25 (1.23–1.27) | 0.95 (0.94–0.97) | 0.77 (0.74–0.80) | 1.20 (1.18–1.23) | 0.90 (0.88–0.92) | 0.68 (0.65–0.71) |
| 11^th^ | 1.32 (1.30–1.34) | 0.95 (0.93–0.97) | 0.70 (0.67–0.72) | 1.26 (1.24–1.28) | 0.89 (0.87–0.91) | 0.61 (0.58–0.64) |
| 12^th^ | 1.45 (1.42–1.47) | 0.91 (0.89–0.93) | 0.63 (0.60–0.65) | 1.38 (1.36–1.41) | 0.84 (0.82–0.86) | 0.53 (0.51–0.56) |
| Economic status |  |  |  |  |  |  |
| High | referent | referent | referent | referent | referent | referent |
| High middle | 0.92 (0.91–0.94) | 0.88 (0.86–0.90) | 0.63 (0.60–0.65) | 0.93 (0.91–0.95) | 0.91 (0.88–0.93) | 0.68 (0.64–0.71) |
| Middle | 0.89 (0.87–0.90) | 0.87 (0.85–0.89) | 0.58 (0.56–0.60) | 0.91 (0.89–0.93) | 0.91 (0.88–0.93) | 0.62 (0.60–0.65) |
| Low middle | 1.26 (1.23–1.28) | 1.36 (1.33–1.40) | 0.89 (0.85–0.93) | 1.28 (1.25–1.31) | 1.42 (1.38–1.47) | 0.94 (0.89–0.99) |
| Low | 1.80 (1.75–1.85) | 2.13 (2.07–2.20) | 1.77 (1.68–1.85) | 1.85 (1.79–1.91) | 2.20 (2.11–2.28) | 1.76 (1.65–1.88) |
| Academic achievement | |  |  |  |  |  |
| High | referent | referent | referent | referent | referent | referent |
| High middle | 1.08 (1.06–1.09) | 0.98 (0.96–1.01) | 0.86 (0.82–0.89) | 1.11 (1.09–1.14) | 1.02 (1.00–1.04) | 0.96 (0.91–1.00) |
| Middle | 1.16 (1.14–1.18) | 1.01 (0.99–1.03) | 0.99 (0.95–1.03) | 1.22 (1.20–1.25) | 1.07 (1.04–1.09) | 1.10 (1.05–1.16) |
| Low middle | 1.39 (1.37–1.42) | 1.24 (1.22–1.27) | 1.31 (1.26–1.36) | 1.51 (1.48–1.53) | 1.35 (1.32–1.38) | 1.51 (1.45–1.59) |
| Low | 1.71 (1.68–1.74) | 1.55 (1.51–1.59) | 1.97 (1.89–2.06) | 1.89 (1.85–1.93) | 1.71 (1.67–1.76) | 2.30 (2.19–2.42) |
| Paternal educational level | |  |  |  |  |  |
| >12 years |  |  |  | referent | referent | referent |
| 12 years |  |  |  | 0.93 (0.92–0.94) | 0.93 (0.91–0.94) | 0.98 (0.95–1.01) |
| <12 years |  |  |  | 1.00 (0.98–1.03) | 1.03 (1.00–1.07) | 1.15 (1.09–1.22) |
| Maternal educational level | |  |  |  |  |  |
| <12 years |  |  |  | referent | referent | referent |
| 12 years |  |  |  | 1.02 (1.00–1.03) | 1.00 (0.99–1.02) | 0.99 (0.96–1.03) |
| >12 years |  |  |  | 1.13 (1.10–1.16) | 1.16 (1.12–1.19) | 1.15 (1.08–1.22) |

**Table S6.** Distribution of perceived economic status and school achievement in each year.

| Unweighted n (%) | 2006 | 2007 | 2008 | 2009 | 2010 | 2011 | 2012 | 2013 | 2014 | 2015 | 2016 | 2017 | 2018 | total |
| --- | --- | --- | --- | --- | --- | --- | --- | --- | --- | --- | --- | --- | --- | --- |
| **Economic status** | | | | | | | | | | | | | | |
| High | 5264 | 4237 | 4640 | 4357 | 4778 | 4777 | 4783 | 5222 | 5612 | 6214 | 6247 | 6713 | 6526 | 69370 |
|  | 7.4% | 5.7% | 6.2% | 5.8% | 6.5% | 6.3% | 6.4% | 7.2% | 7.8% | 9.1% | 9.5% | 10.8% | 10.9% | 7.5% |
| High-middle | 15678 | 16042 | 16161 | 15893 | 16294 | 17654 | 17499 | 17525 | 18333 | 18316 | 17997 | 18089 | 17681 | 223162 |
|  | 22.0% | 21.5% | 21.5% | 21.2% | 22.2% | 23.3% | 23.6% | 24.2% | 25.4% | 26.9% | 27.5% | 29.0% | 29.4% | 24.3% |
| Middle | 33870 | 35523 | 35770 | 35449 | 34253 | 35762 | 34884 | 34494 | 35040 | 31962 | 31056 | 28582 | 27808 | 434453 |
|  | 47.4% | 47.6% | 47.5% | 47.2% | 46.8% | 47.3% | 47.0% | 47.6% | 48.6% | 47.0% | 47.4% | 45.9% | 46.3% | 47.2% |
| Low-middle | 12472 | 14226 | 13692 | 14240 | 13390 | 13549 | 13213 | 11806 | 10353 | 9330 | 8324 | 7299 | 6582 | 148476 |
|  | 17.5% | 19.0% | 18.2% | 19.0% | 18.3% | 17.9% | 17.8% | 16.3% | 14.4% | 13.7% | 12.7% | 11.7% | 11.0% | 16.1% |
| Low | 4120 | 4670 | 4975 | 5127 | 4523 | 3901 | 3807 | 3388 | 2722 | 2221 | 1904 | 1593 | 1443 | 44394 |
|  | 5.8% | 6.3% | 6.6% | 6.8% | 6.2% | 5.2% | 5.1% | 4.7% | 3.8% | 3.3% | 2.9% | 2.6% | 2.4% | 4.8% |
| **School achievement** | | | | | | | | | | | | | | |
| High | 9753 | 8912 | 8669 | 8412 | 8359 | 8313 | 7920 | 7942 | 8849 | 8615 | 8689 | 8528 | 8069 | 111030 |
|  | 13.7% | 11.9% | 11.5% | 11.2% | 11.4% | 11.0% | 10.7% | 11.0% | 12.3% | 12.7% | 13.3% | 13.7% | 13.4% | 12.1% |
| High-middle | 18100 | 18489 | 17224 | 17583 | 17592 | 18253 | 17602 | 17053 | 17874 | 17075 | 16550 | 15996 | 15351 | 224742 |
|  | 25.3% | 24.8% | 22.9% | 23.4% | 24.0% | 24.1% | 23.7% | 23.5% | 24.8% | 25.1% | 25.3% | 25.7% | 25.6% | 24.4% |
| Middle | 19478 | 19896 | 20578 | 20219 | 19651 | 20375 | 19854 | 20148 | 20162 | 18952 | 18626 | 17810 | 17526 | 253275 |
|  | 27.3% | 26.6% | 27.4% | 26.9% | 26.8% | 26.9% | 26.8% | 27.8% | 28.0% | 27.9% | 28.4% | 28.6% | 29.2% | 27.5% |
| Low-middle | 16494 | 18525 | 19341 | 19138 | 18704 | 19335 | 18941 | 18107 | 17304 | 15994 | 15076 | 13818 | 13249 | 224026 |
|  | 23.1% | 24.8% | 25.7% | 25.5% | 25.5% | 25.6% | 25.5% | 25.0% | 24.0% | 23.5% | 23.0% | 22.2% | 22.1% | 24.4% |
| Low | 7579 | 8876 | 9426 | 9714 | 8932 | 9367 | 9869 | 9185 | 7871 | 7407 | 6587 | 6124 | 5845 | 106782 |
|  | 10.6% | 11.9% | 12.5% | 12.9% | 12.2% | 12.4% | 13.3% | 12.7% | 10.9% | 10.9% | 10.1% | 9.8% | 9.7% | 11.6% |

**Table S7.** Social index of South Korea during the study year.

|  | 2006 | 2007 | 2008 | 2009 | 2010 | 2011 | 2012 | 2013 | 2014 | 2015 | 2016 | 2017 | 2018 |
| --- | --- | --- | --- | --- | --- | --- | --- | --- | --- | --- | --- | --- | --- |
| Gini Index | 0.306 | 0.312 | 0.314 | 0.314 | 0.310 | 0.311 | 0.307 | 0.302 | 0.302 | 0.295 | 0.304 |  |  |
| Admission to tertiary education service | 82.1% | 82.8% | 83.8% | 81.9% | 78.9% | 72.5% | 71.3% | 70.7% | 70.9% | 70.8% | 69.8% | 68.9% | 69.7% |
| Completed suicides (n) | 201 | 260 | 269 | 369 | 292 | 317 | 289 | 271 | 243 | 214 | 251 | 219 | 251 |
