# Supplementary material for: Investigation of the Trend in Adolescent Mental Health and its Related Social Factors: A Multi-Year Cross-Sectional Study For 13 Years
Source: Int J Environ Res Public Health. 2020 Jul 27;17(15):5405. doi: 10.3390/ijerph17155405 (PMC7432642; doi:10.3390/ijerph17155405)
Supplement: Supplementary file 1 [file ijerph-17-05405-s001.zip › ijerph-850326-supplementary.pdf]

Supplementary Materials: Investigation of the Trend in Adolescent Mental Health and its Related Social Factors: A Multi-Year Cross-Sectional Study For 13 Years

Table S1. The number of participants by academic years with the regional distribution.

	Total (N)	Total (%)	2006	2007	2008	2009	2010	2011	2012	2013	2014	2015	2016	2017	2018
Gangwon-do	36762	4.0%	3402	3352	3520	3553	3447	2722	2486	2457	2669	2449	2224	2219	2262
Gyeonggi-do	160025	17.4%	8291	8761	9332	9430	9044	14427	15658	15319	15158	14352	13990	13465	12798
Gyeongsangnam-do	60252	6.6%	4848	5014	4849	4933	4810	4733	4920	4744	4737	4581	4132	4067	3884
Gyeongsangbuk-do	50996	5.5%	4266	4497	4589	4589	4515	4028	3755	3924	3765	3557	3335	3112	3064
Gwangju	43007	4.7%	3846	4076	4011	4110	4024	3309	3149	3002	3072	2850	2676	2441	2441
Daegu	55376	6.0%	4953	5163	5053	5063	5030	4439	4312	4151	3952	3614	3292	3288	3066
Daejeon	41380	4.5%	3798	3870	4011	3948	3874	3003	2975	2947	2966	2656	2682	2377	2273
Busan	61936	6.7%	5195	5530	5501	5468	5303	5157	4939	4649	4436	4209	3979	3752	3818
Seoul	122614	13.3%	7855	8402	8077	8207	7848	11355	11373	11165	10997	9710	9567	9287	8771
Sejong	3823	0.4%	0	0	0	0	0	0	0	0	0	935	1012	932	944
Ulsan	36824	4.0%	3561	3723	3665	3635	3478	2913	2518	2486	2449	2218	2114	2121	1943
Incheon	56853	6.2%	4879	5122	5240	5126	5008	4516	4302	4140	4120	3847	3724	3431	3398
Jeollanam-do	40673	4.4%	3564	3661	3581	3381	3381	3434	2906	2871	3111	2925	2820	2583	2455
Jeollabuk-do	42015	4.6%	3336	3548	3757	3799	3778	3382	3133	3183	3012	2931	3060	2611	2485
Jeju-do	25115	2.7%	2562	2638	2485	2336	2329	1795	1743	1560	1664	1597	1478	1476	1452
Chungcheongnam-do	43493	4.7%	3624	3740	3910	3852	3869	3457	3264	3112	3216	3095	2990	2745	2619
Chungcheongbuk-do	38711	4.2%	3424	3601	3657	3636	3500	2973	2753	2725	2736	2517	2453	2369	2367
Total	919855	100.0%	71404	74698	75238	75066	73238	75643	74186	72435	72060	68043	65528	62276	60040

Table S2. General perceived subjective happiness of participants at each year.

**Unweighted** n (%)	2006	2007	2008	2009	2010	2011	2012	2013	2014	2015	2016	2017
Very happy	12064	11519	12451	12590	12993	13629	13771	15410	17943	18276	18992	18573
	16.9%	15.4%	16.5%	16.8%	17.7%	18.0%	18.6%	21.3%	24.9%	26.9%	29.0%	29.8%
Slightly happy	25705	26397	27039	26882	26732	29037	27941	26717	27853	26473	24964	23564
	36.0%	35.3%	35.9%	35.8%	36.5%	38.4%	37.7%	36.9%	38.7%	38.9%	38.1%	37.8%
Neutral	23646	25669	25109	25387	23601	23387	22725	21943	19908	17922	16743	15309
	33.1%	34.4%	33.4%	33.8%	32.2%	30.9%	30.6%	30.3%	27.6%	26.3%	25.6%	24.6%
Slightly unhappy	8214	9081	8736	8389	8221	8112	8310	7095	5403	4532	4102	4041
	11.5%	12.2%	11.6%	11.2%	11.2%	10.7%	11.2%	9.8%	7.5%	6.7%	6.3%	6.5%
Very unhappy	1775	2032	1903	1818	1691	1478	1439	1270	953	840	727	789
	2.5%	2.7%	2.5%	2.4%	2.3%	2.0%	1.9%	1.8%	1.3%	1.2%	1.1%	1.3%

Table S3. Distribution of depressive episode, suicidal ideation, and suicidal attempt.

Unweighted n (%)	2006	2007	2008	2009	2010	2011	2012	2013	2014	2015	2016	2017	2018
Depressive episode													
Yes	29498	30951	29202	28273	27373	25161	22745	22430	19174	15894	16535	15612	16208
	41.3%	41.4%	38.8%	37.7%	37.4%	33.3%	30.7%	31.0%	26.6%	23.4%	25.2%	25.1%	27.0%
No	41903	43747	46036	46793	45865	50482	51441	50005	52886	52149	48993	46664	43832
	58.7%	58.6%	61.2%	62.3%	62.6%	66.7%	69.3%	69.0%	73.4%	76.6%	74.8%	74.9%	73.0%
Suicidal ideation													
Yes	16397	17783	14259	14458	14011	14875	13635	12070	9438	7862	7845	7584	7976
	23.0%	23.8%	19.0%	19.3%	19.1%	19.7%	18.4%	16.7%	13.1%	11.6%	12.0%	12.2%	13.3%
No	55004	56915	60979	60608	59227	60768	60551	60365	62622	60181	57683	54692	52064
	77.0%	76.2%	81.0%	80.7%	80.9%	80.3%	81.6%	83.3%	86.9%	88.4%	88.0%	87.8%	86.7%
Suicidal attempt													
Yes	3817	4460	3649	3513	3616	3304	3018	3021	2104	1662	1530	1634	1873
	5.3%	6.0%	4.8%	4.7%	4.9%	4.4%	4.1%	4.2%	2.9%	2.4%	2.3%	2.6%	3.1%
No	67587	70238	71589	71553	69622	72339	71168	69414	69956	66381	63998	60642	58167
	94.7%	94.0%	95.2%	95.3%	95.1%	95.6%	95.9%	95.8%	97.1%	97.6%	97.7%	97.4%	96.9%

Table S4. Mean scores of subjective happiness according to the demographic factors (mean; SD).

Demographic variables						
	High	High-middle	Middle	Low-middle	Low	
Economic status	4.07 (1.00)	3.87 (0.92)	3.63 (0.92)	3.36 (0.98)	3.09 (1.14)	
Academic achievement	3.91 (0.97)	3.77 (0.93)	3.68 (0.93)	3.53 (0.97)	3.33 (1.07)	
Sex	Male	Female				
	3.71 (0.98)	3.58 (0.97)				
Grade	7th	8th	9th	10th	11th	12th
	3.81 (0.99)	3.70 (0.99)	3.67 (0.97)	3.60 (0.97)	3.55 (0.95)	3.56 (0.96)
Parental educational level	<12 years	12 years	>12 years			
Paternal educational level	3.46 (1.00)	3.61 (0.96)	3.73 (0.97)			
Maternal educational level	3.46 (0.98)	3.61 (0.96)	3.76 (0.97)			

Table S5. Logistic regression analysis for depressive episode, suicidal ideation, and suicidal attempt with the demographic variables.

	Model 1 (n = 919855)			Model 2 (n = 694753)		
	Depressive episode	Suicidal ideation	Suicidal attempt	Depressive episode	Suicidal ideation	Suicidal attempt
Sex						
Male	referent	referent	referent	referent	referent	referent
Female	1.63 (1.61–1.64)	1.71 (1.69–1.73)	1.77 (1.74–1.81)	1.58 (1.57–1.6)	1.67 (1.65–1.70)	1.75 (1.70–1.79)
Grade						
7th	referent	referent	referent	referent	referent	referent
8th	1.10 (1.09–1.12)	1.05 (1.03–1.07)	1.00 (0.96–1.03)	1.08 (1.06–1.11)	1.01 (0.99–1.03)	0.92 (0.89–0.96)
9th	1.20 (1.18–1.22)	1.04 (1.02–1.06)	0.92 (0.89–0.95)	1.18 (1.16–1.20)	1.00 (0.98–1.02)	0.86 (0.82–0.89)
10th	1.25 (1.23–1.27)	0.95 (0.94–0.97)	0.77 (0.74–0.80)	1.20 (1.18–1.23)	0.90 (0.88–0.92)	0.68 (0.65–0.71)
11th	1.32 (1.30–1.34)	0.95 (0.93–0.97)	0.70 (0.67–0.72)	1.26 (1.24–1.28)	0.89 (0.87–0.91)	0.61 (0.58–0.64)
12th	1.45 (1.42–1.47)	0.91 (0.89–0.93)	0.63 (0.60–0.65)	1.38 (1.36–1.41)	0.84 (0.82–0.86)	0.53 (0.51–0.56)
Economic status						
High	referent	referent	referent	referent	referent	referent
High middle	0.92 (0.91–0.94)	0.88 (0.86–0.90)	0.63 (0.60–0.65)	0.93 (0.91–0.95)	0.91 (0.88–0.93)	0.68 (0.64–0.71)
Middle	0.89 (0.87–0.90)	0.87 (0.85–0.89)	0.58 (0.56–0.60)	0.91 (0.89–0.93)	0.91 (0.88–0.93)	0.62 (0.60–0.65)
Low middle	1.26 (1.23–1.28)	1.36 (1.33–1.40)	0.89 (0.85–0.93)	1.28 (1.25–1.31)	1.42 (1.38–1.47)	0.94 (0.89–0.99)
Low	1.80 (1.75–1.85)	2.13 (2.07–2.20)	1.77 (1.68–1.85)	1.85 (1.79–1.91)	2.20 (2.11–2.28)	1.76 (1.65–1.88)
Academic achievement						
High	referent	referent	referent	referent	referent	referent
High middle	1.08 (1.06–1.09)	0.98 (0.96–1.01)	0.86 (0.82–0.89)	1.11 (1.09–1.14)	1.02 (1.00–1.04)	0.96 (0.91–1.00)
Middle	1.16 (1.14–1.18)	1.01 (0.99–1.03)	0.99 (0.95–1.03)	1.22 (1.20–1.25)	1.07 (1.04–1.09)	1.10 (1.05–1.16)
Low middle	1.39 (1.37–1.42)	1.24 (1.22–1.27)	1.31 (1.26–1.36)	1.51 (1.48–1.53)	1.35 (1.32–1.38)	1.51 (1.45–1.59)
Low	1.71 (1.68–1.74)	1.55 (1.51–1.59)	1.97 (1.89–2.06)	1.89 (1.85–1.93)	1.71 (1.67–1.76)	2.30 (2.19–2.42)
Paternal educational level						
>12 years				referent	referent	referent
12 years				0.93 (0.92–0.94)	0.93 (0.91–0.94)	0.98 (0.95–1.01)
<12 years				1.00 (0.98–1.03)	1.03 (1.00–1.07)	1.15 (1.09–1.22)
Maternal educational level						
<12 years				referent	referent	referent
12 years				1.02 (1.00–1.03)	1.00 (0.99–1.02)	0.99 (0.96–1.03)
>12 years				1.13 (1.10–1.16)	1.16 (1.12–1.19)	1.15 (1.08–1.22)

Table S6. Distribution of perceived economic status and school achievement in each year.

Unweighted n (%)	2006	2007	2008	2009	2010	2011	2012	2013	2014	2015	2016	2017	2018	total
**Economic status**														
High	5264	4237	4640	4357	4778	4777	4783	5222	5612	6214	6247	6713	6526	69370
	7.4%	5.7%	6.2%	5.8%	6.5%	6.3%	6.4%	7.2%	7.8%	9.1%	9.5%	10.8%	10.9%	7.5%
High-middle	15678	16042	16161	15893	16294	17654	17499	17525	18333	18316	17997	18089	17681	223162
	22.0%	21.5%	21.5%	21.2%	22.2%	23.3%	23.6%	24.2%	25.4%	26.9%	27.5%	29.0%	29.4%	24.3%
Middle	33870	35523	35770	35449	34253	35762	34884	34494	35040	31962	31056	28582	27808	434453
	47.4%	47.6%	47.5%	47.2%	46.8%	47.3%	47.0%	47.6%	48.6%	47.0%	47.4%	45.9%	46.3%	47.2%
Low-middle	12472	14226	13692	14240	13390	13549	13213	11806	10353	9330	8324	7299	6582	148476
	17.5%	19.0%	18.2%	19.0%	18.3%	17.9%	17.8%	16.3%	14.4%	13.7%	12.7%	11.7%	11.0%	16.1%
Low	4120	4670	4975	5127	4523	3901	3807	3388	2722	2221	1904	1593	1443	44394
	5.8%	6.3%	6.6%	6.8%	6.2%	5.2%	5.1%	4.7%	3.8%	3.3%	2.9%	2.6%	2.4%	4.8%
**School achievement**														
High	9753	8912	8669	8412	8359	8313	7920	7942	8849	8615	8689	8528	8069	111030
	13.7%	11.9%	11.5%	11.2%	11.4%	11.0%	10.7%	11.0%	12.3%	12.7%	13.3%	13.7%	13.4%	12.1%
High-middle	18100	18489	17224	17583	17592	18253	17602	17053	17874	17075	16550	15996	15351	224742
	25.3%	24.8%	22.9%	23.4%	24.0%	24.1%	23.7%	23.5%	24.8%	25.1%	25.3%	25.7%	25.6%	24.4%
Middle	19478	19896	20578	20219	19651	20375	19854	20148	20162	18952	18626	17810	17526	253275
	27.3%	26.6%	27.4%	26.9%	26.8%	26.9%	26.8%	27.8%	28.0%	27.9%	28.4%	28.6%	29.2%	27.5%
Low-middle	16494	18525	19341	19138	18704	19335	18941	18107	17304	15994	15076	13818	13249	224026
	23.1%	24.8%	25.7%	25.5%	25.5%	25.6%	25.5%	25.0%	24.0%	23.5%	23.0%	22.2%	22.1%	24.4%
Low	7579	8876	9426	9714	8932	9367	9869	9185	7871	7407	6587	6124	5845	106782
	10.6%	11.9%	12.5%	12.9%	12.2%	12.4%	13.3%	12.7%	10.9%	10.9%	10.1%	9.8%	9.7%	11.6%

Table S7. Social index of South Korea during the study year.

	2006	2007	2008	2009	2010	2011	2012	2013	2014	2015	2016	2017	2018
Gini Index	0.306	0.312	0.314	0.314	0.310	0.311	0.307	0.302	0.302	0.295	0.304		
Admission to tertiary education service	82.1%	82.8%	83.8%	81.9%	78.9%	72.5%	71.3%	70.7%	70.9%	70.8%	69.8%	68.9%	69.7%
Completed suicides (n)	201	260	269	369	292	317	289	271	243	214	251	219	251
